# Supplementary figures and images for: Endoplasmic Reticulum Associated Degradation of Spinocerebellar Ataxia-Related CD10 Cysteine Mutant
Source: Int J Mol Sci. 2020 Jun 14;21(12):4237. doi: 10.3390/ijms21124237 (PMC7352294; doi:10.3390/ijms21124237)

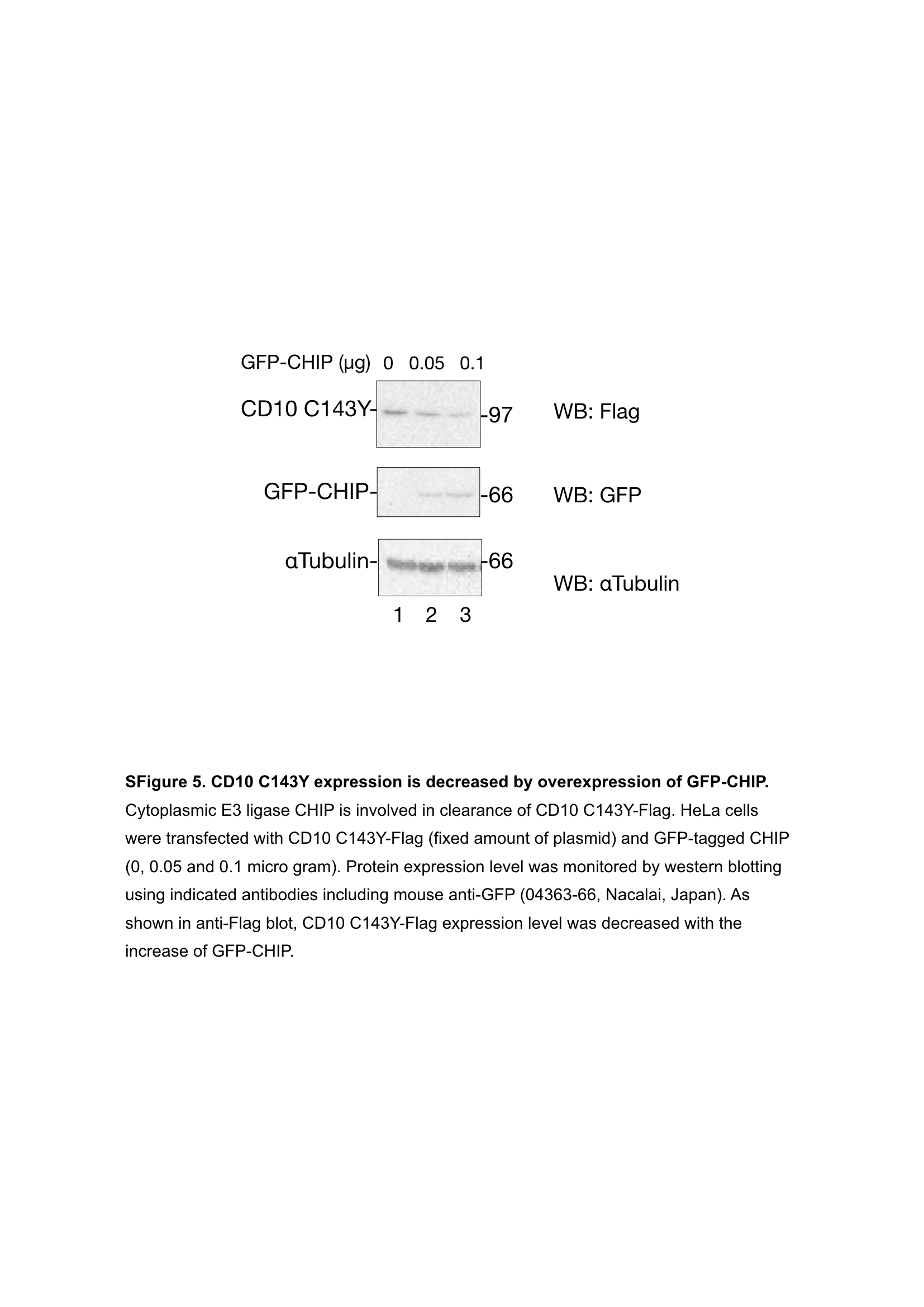

Supplement: Supplementary file 1 [file ijms-21-04237-s001.zip › sFig5.tiff]

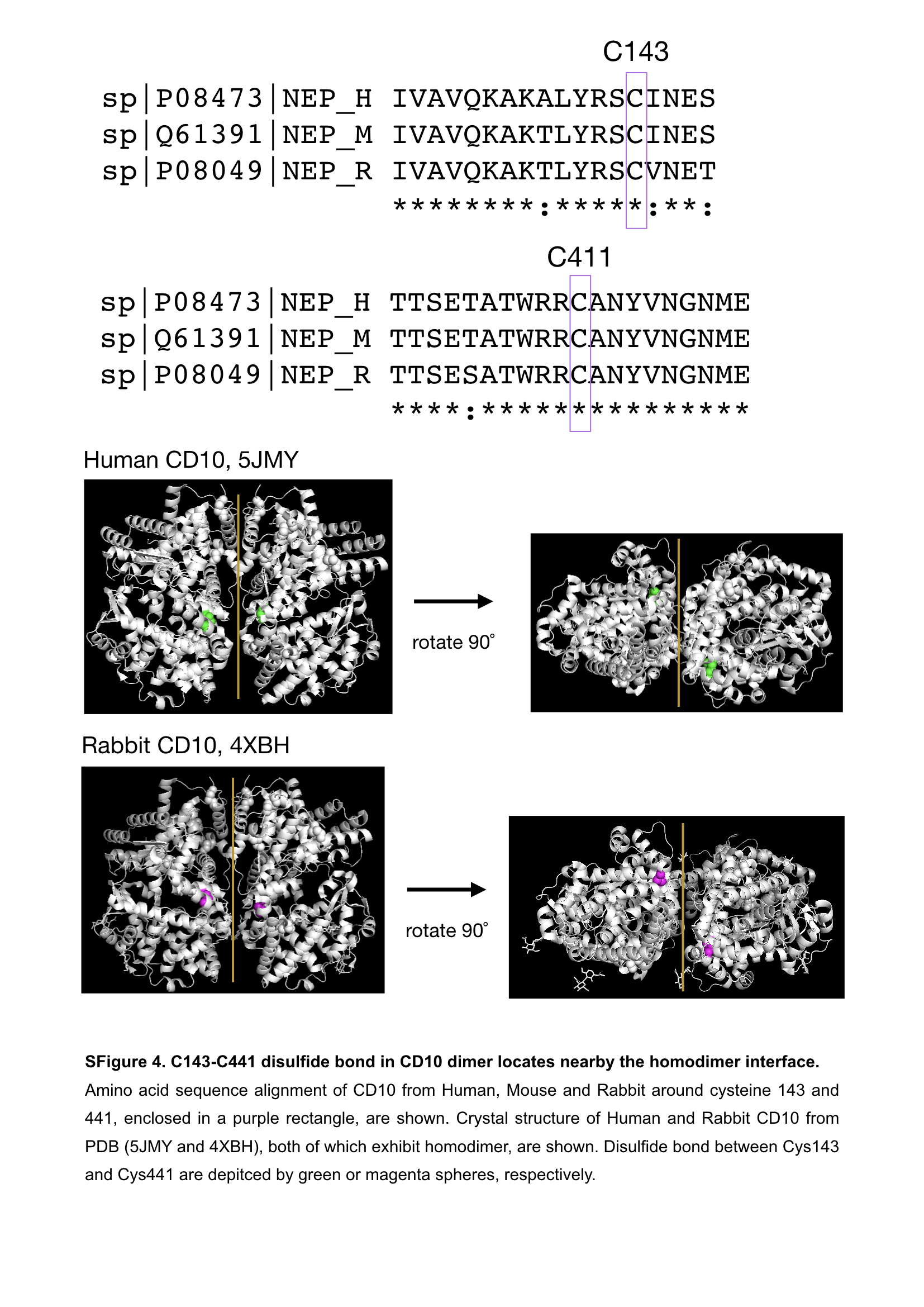

Supplement: Supplementary file 1 [file ijms-21-04237-s001.zip › sFig4.tiff]

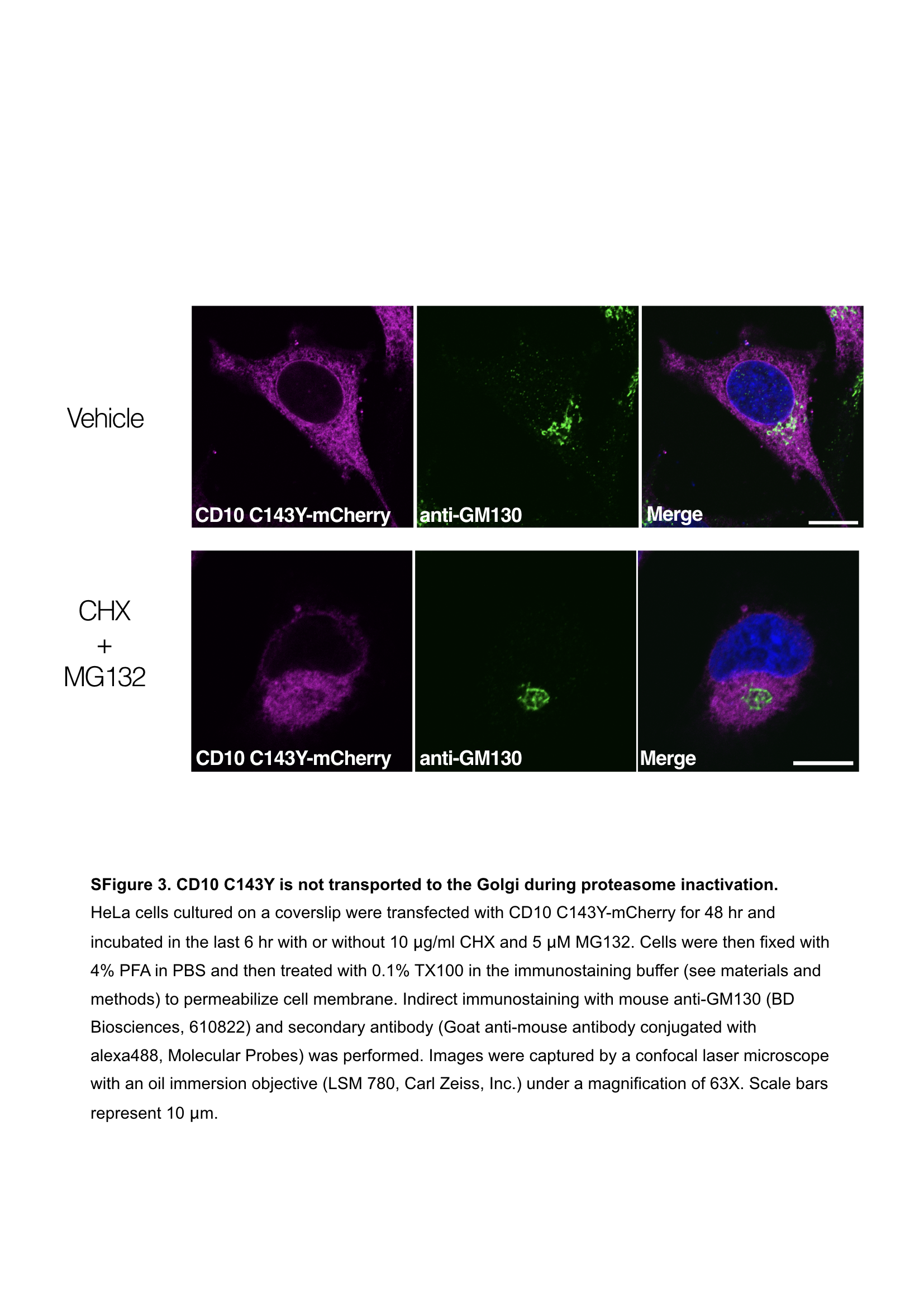

Supplement: Supplementary file 1 [file ijms-21-04237-s001.zip › sFig3.tiff]

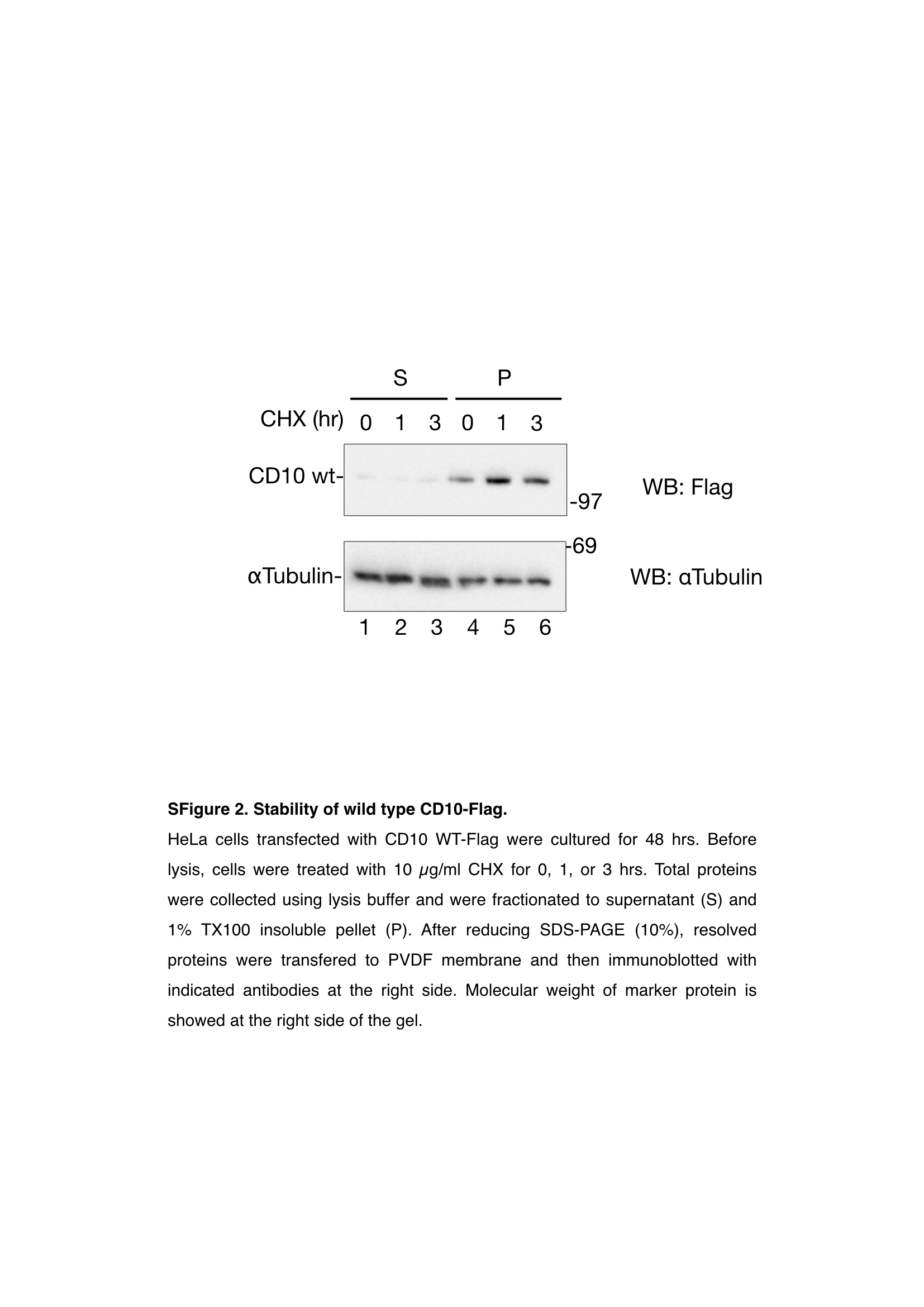

Supplement: Supplementary file 1 [file ijms-21-04237-s001.zip › sFig2.tiff]

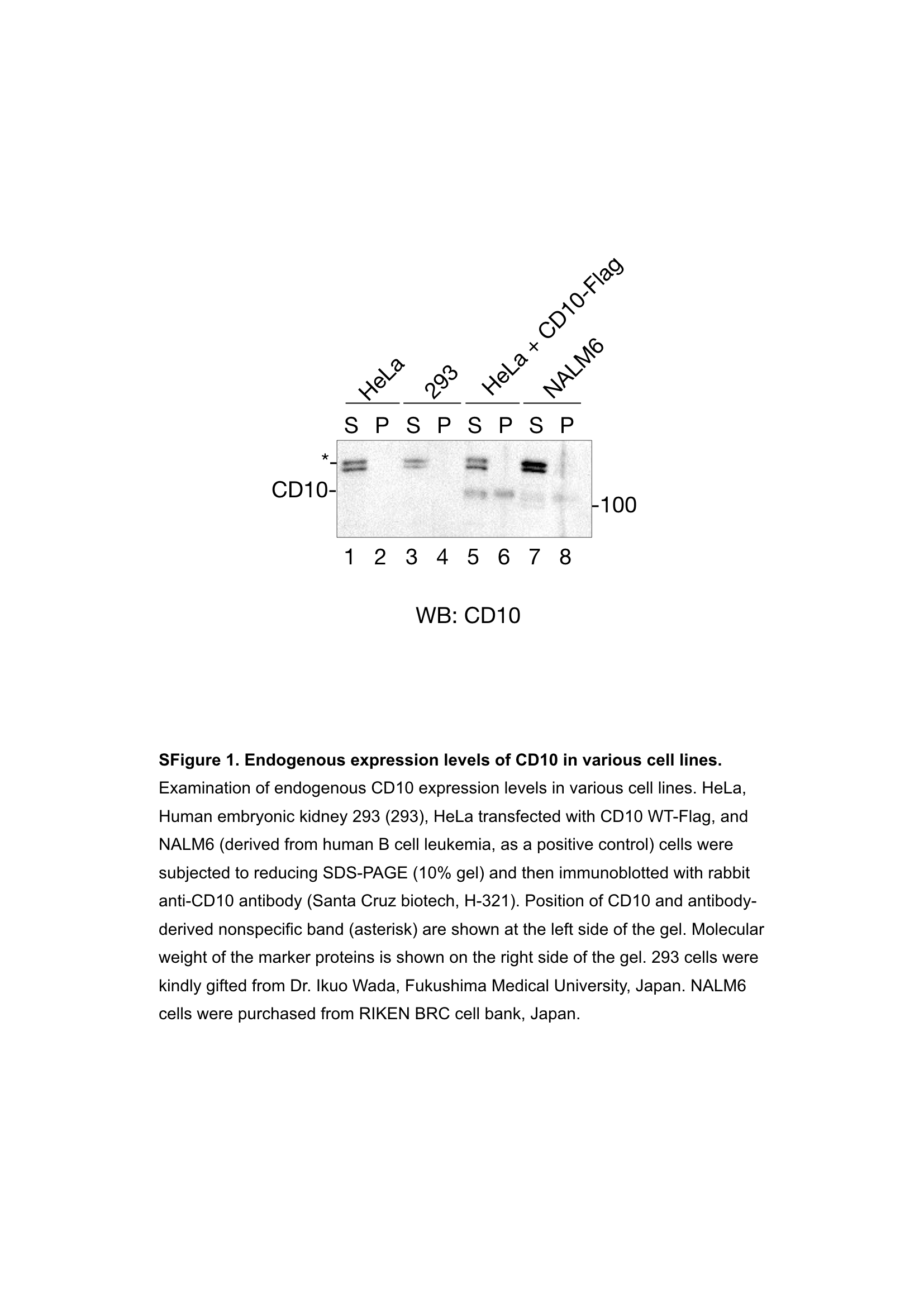

Supplement: Supplementary file 1 [file ijms-21-04237-s001.zip › sFig1.tiff]

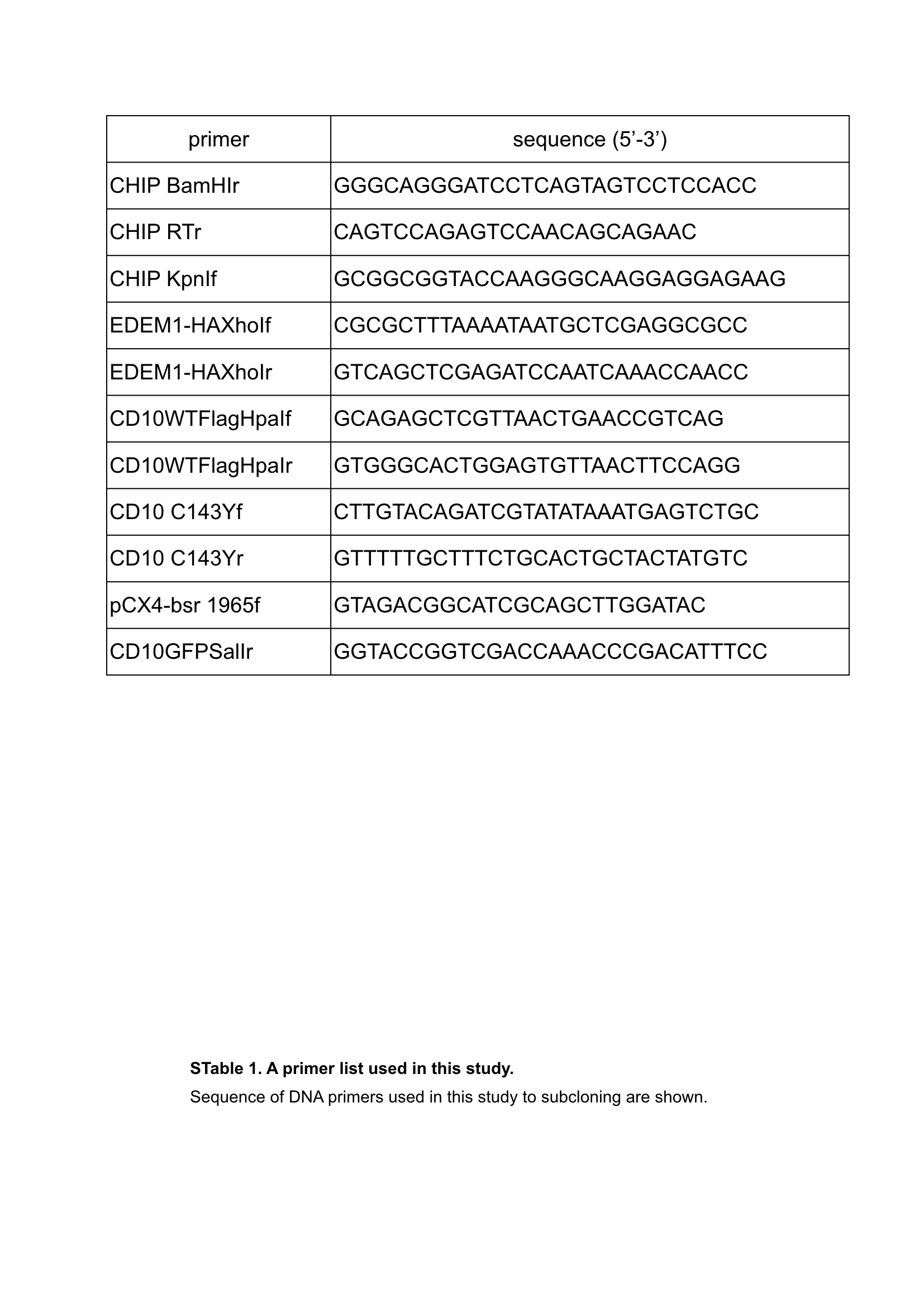

Supplement: Supplementary file 1 [file ijms-21-04237-s001.zip › sTable1.tiff]
